# Supplementary material for: Phytoalexin sakuranetin attenuates endocytosis and enhances resistance to rice blast
Source: Nat Commun. 2024 Apr 23;15:3437. doi: 10.1038/s41467-024-47746-y (PMC11039461; doi:10.1038/s41467-024-47746-y)
Supplement: Supplementary file 1 — Supplemental materials [file 41467_2024_47746_MOESM1_ESM.pdf]

Supplementary Information

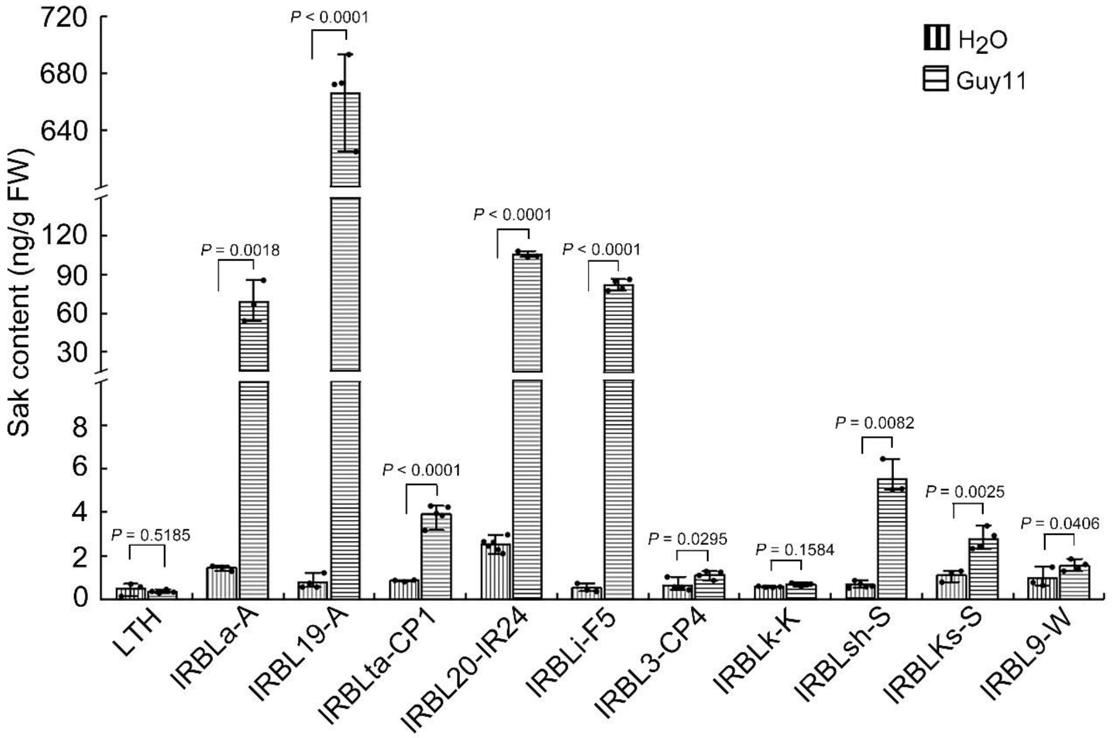

Figure. 1. The sakuranetin levels in the leaves of rice near-isogenic lines

Sakuranetin levels in the leaves of 3-week old rice seedlings of LTH and near-isogenic lines IRBLa-A, IRBL19-A, IRBLta-CP1, IRBL20-IR24, IRBLi-F5, IRBL3-CP4, IRBLk-K, IRBLsh-S, IRBLKs-S and IRBL9-W ($n_{LTH} = 3$, $n_{IRBLa\text{-}A} = 3$, $n_{IRBL19\text{-}A} = 4$, $n_{IRBLta\text{-}CP1} = 3$, $n_{IRBL20\text{-}IR24} = 6$, $n_{IRBLi\text{-}F5} = 3$, $n_{IRBL3\text{-}CP4} = 4$, $n_{IRBLk\text{-}K} = 4$, $n_{IRBLsh\text{-}S} = 5$, $n_{IRBLKs\text{-}S} = 3$, $n_{IRBL9\text{-}W} = 3$) inoculated with fungus *M. oryzae* strain Guy11 ($n_{LTH} = 4$, $n_{IRBLa\text{-}A} = 3$, $n_{IRBL19\text{-}A} = 4$, $n_{IRBLta\text{-}CP1} = 5$, $n_{IRBL20\text{-}IR24} = 3$, $n_{IRBLi\text{-}F5} = 4$, $n_{IRBL3\text{-}CP4} = 4$, $n_{IRBLk\text{-}K} = 3$, $n_{IRBLsh\text{-}S} = 3$, $n_{IRBLKs\text{-}S} = 4$, $n_{IRBL9\text{-}W} = 4$). Sak = sakuranetin. FW = fresh weight. Data are means ± SE; *P* values were generated using an independent-samples two-sided Student's *t*-test

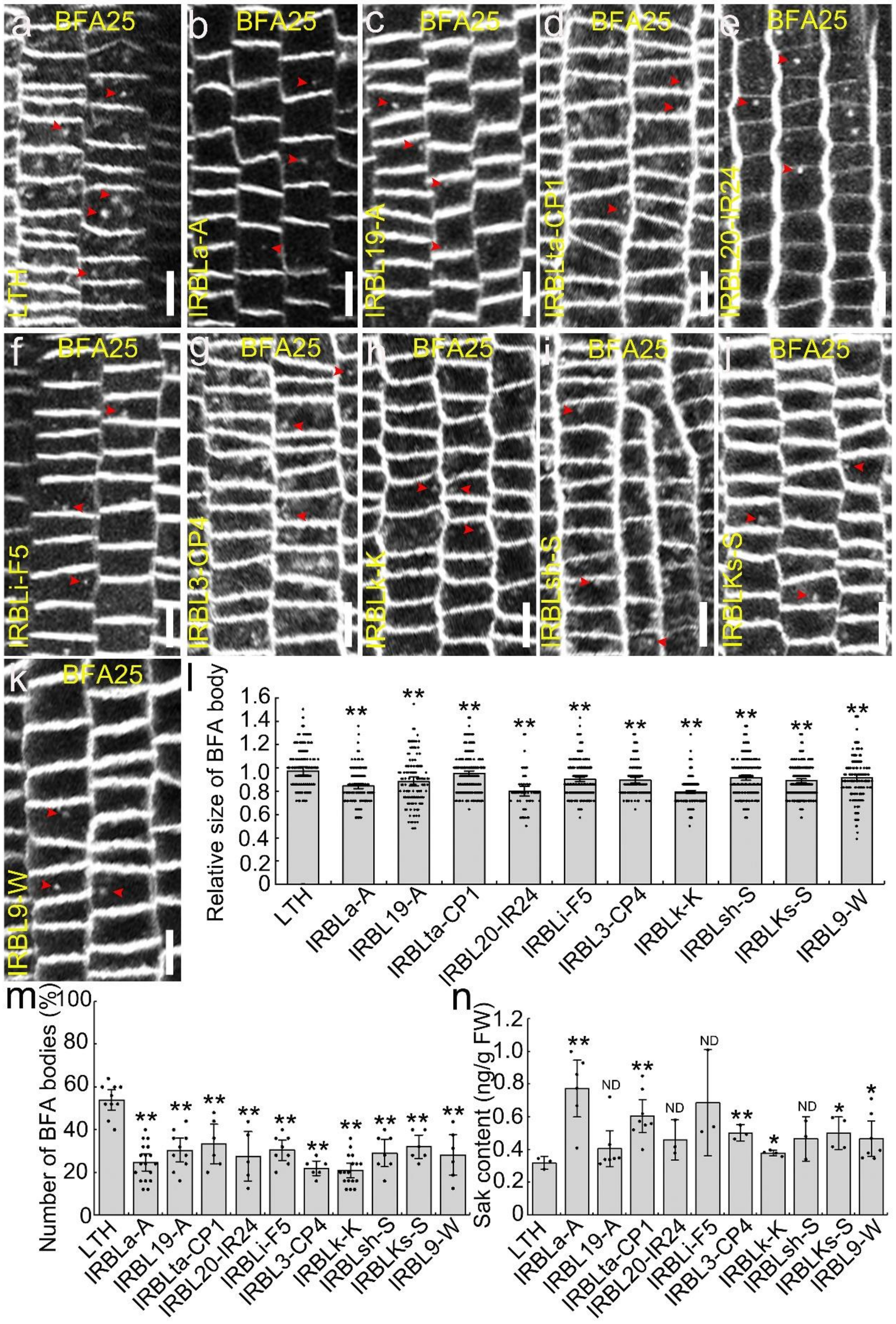

Figure. 2. Endocytosis of plasma membrane of root epidermal cells is attenuated in rice near-isogenic lines

(a-k) The root epidermal cells of 6-day old rice LTH (a), and near-isogenic lines IRBLa-A (b), IRBL19-A (c), IRBLta-CP1 (d), IRBL20-IR24 (e), IRBLi-F5 (f), IRBL3-CP4 (g), IRBLk-K (h), IRBLsh-S (i), IRBLKs-S (j) and IRBL9-W(k) were treated with 25 μM BFA and labelled with 4 μM FM4-64 for 120 minutes. (l) Quantification of the relative sizes of BFA bodies shown in the images a-k (n_{LTH} = 186, $n_{\mathrm{IRBLa\text{-}A}}$ = 164, $n_{\mathrm{IRBL19\text{-}A}}$ = 123, $n_{\mathrm{IRBLta\text{-}CP1}}$ = 253, $n_{\mathrm{IRBL20\text{-}IR24}}$ = 59, $n_{\mathrm{IRBLi\text{-}F5}}$ = 269, $n_{\mathrm{IRBL3\text{-}CP4}}$ = 126, $n_{\mathrm{IRBLk\text{-}K}}$ = 283, $n_{\mathrm{IRBLsh\text{-}S}}$ = 226, $n_{\mathrm{IRBLKs\text{-}S}}$ = 268, $n_{\mathrm{IRBL9\text{-}W}}$ = 161). (m) Number of BFA bodies in cells shown in the images a-k (n_{LTH} = 10, $n_{\mathrm{IRBLa\text{-}A}}$ = 18, $n_{\mathrm{IRBL19\text{-}A}}$ = 10, $n_{\mathrm{IRBLta\text{-}CP1}}$ = 6, $n_{\mathrm{IRBL20\text{-}IR24}}$ = 5, $n_{\mathrm{IRBLi\text{-}F5}}$ = 8, $n_{\mathrm{IRBL3\text{-}CP4}}$ = 6, $n_{\mathrm{IRBLk\text{-}K}}$ = 17, $n_{\mathrm{IRBLsh\text{-}S}}$ = 7, $n_{\mathrm{IRBLKs\text{-}S}}$ = 6, $n_{\mathrm{IRBL9\text{-}W}}$ = 6). (n) Sakuranetin levels in the roots of 7-day old rice seedlings LTH and near-isogenic lines shown in the images a-k (n_{LTH} = 3 , $n_{\mathrm{IRBLa\text{-}A}}$ = 6, $n_{\mathrm{IRBL19\text{-}A}}$ = 7, $n_{\mathrm{IRBLta\text{-}CP1}}$ = 8, $n_{\mathrm{IRBL20\text{-}IR24}}$ = 3, $n_{\mathrm{IRBLi\text{-}F5}}$ = 3 , $n_{\mathrm{IRBL3\text{-}CP4}}$ = 3 , $n_{\mathrm{IRBLk\text{-}K}}$ = 4, $n_{\mathrm{IRBLsh\text{-}S}}$ = 3, $n_{\mathrm{IRBLKs\text{-}S}}$ = 4, $n_{\mathrm{IRBL9\text{-}W}}$ = 7). LTH = Lijiangxintuanheigu. FM4 = FM4-64. Sak = sakuranetin. FW = fresh weight. Data are means ± SE; *$P < 0.05$, **$P < 0.01$, P values were generated using independent-samples two-sided Student's *t*-test in the images l and m and an independent-samples two-sided SPSS analysis in the image n, Scale bar = 10 μm. The red arrowheads indicate the BFA bodies.

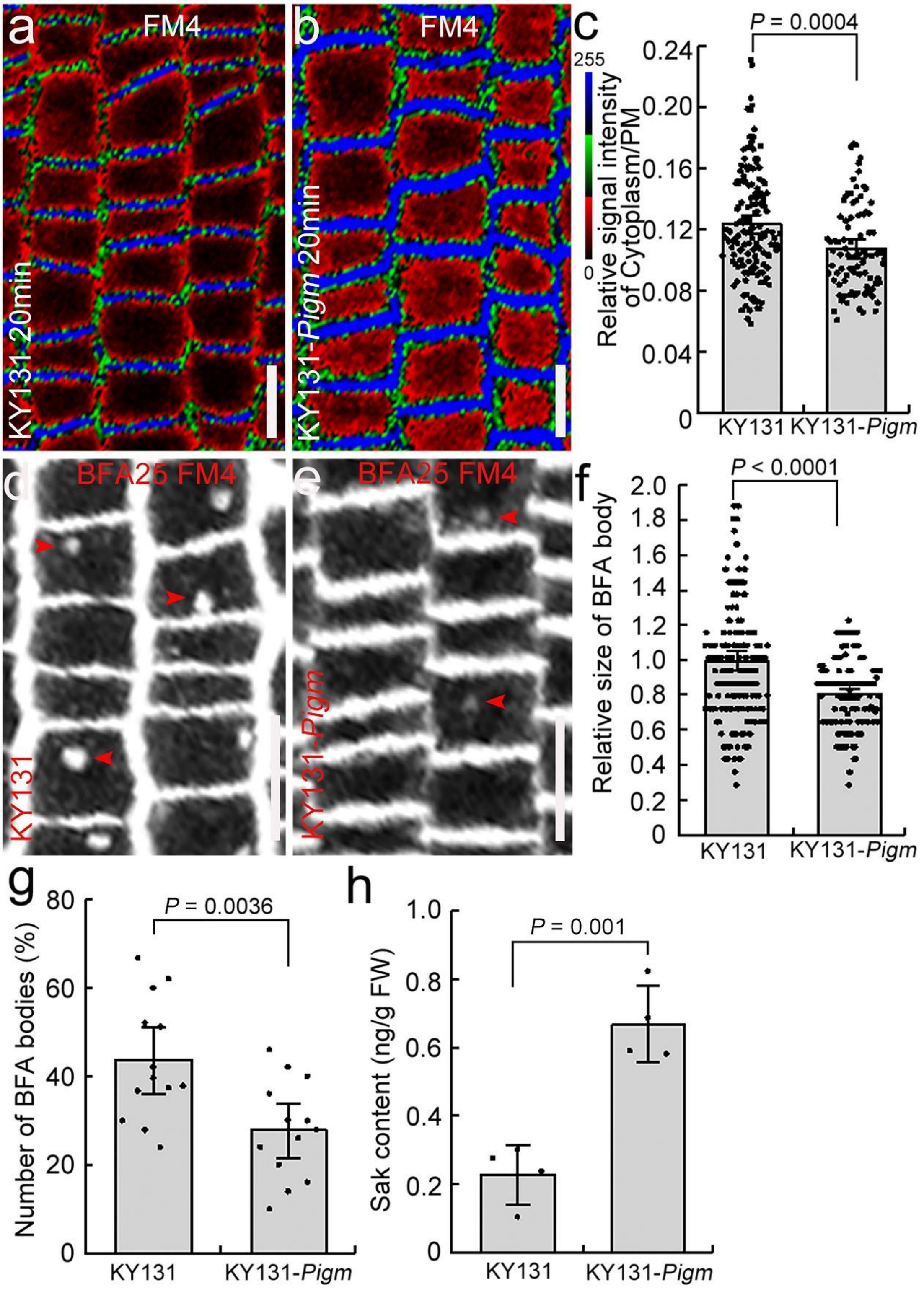

Figure 3. Endocytosis of root cells is attenuated in the resistant rice line KY131-*Pigm*

(a, b) Root epidermal cells of 6-day old rice seedlings KY131 (a) and KY131-*Pigm* (b) were labelled with 4 μM FM4-64 for 90 minutes. (c) Quantification of

fluorescence intensity of PM versus cytoplasm shown in images a-b (n_{KY131} = 150, $n_{\mathrm{KY131}\text{-}Pigm}$ = 90). (d, e) The root epidermal cells of rice seedlings KY131 (d) and KY131-*Pigm* (e) were treated with 25 μM BFA and labelled with 4 μM FM4-64 for 90 minutes. (f) Quantification of relative sizes of BFA bodies shown in images d and e (n_{KY131} = 182, $n_{\mathrm{KY131}\text{-}Pigm}$ = 151). (g) Number of BFA bodies in cells shown in the images d-e (n_{KY131} = 13, $n_{\mathrm{KY131}\text{-}Pigm}$ = 13). (h) Sakuranetin levels in the roots of 7-day old rice lines KY131 and KY131-*Pigm* shown in the images a and b (n_{KY131} = 4, $n_{\mathrm{KY131}\text{-}Pigm}$ = 4). The relative fluorescence intensity is color-coded: red, low; green, medium; and blue, high fluorescence. PM = plasma membrane, FM4 = FM4-64. Sak = sakuranetin. FW = fresh weight. Data are means ± SE; *P* values were generated using an independent-samples two-sided Student's *t*-test. Scale bar = 10 μm. The red arrowheads indicate the BFA bodies.

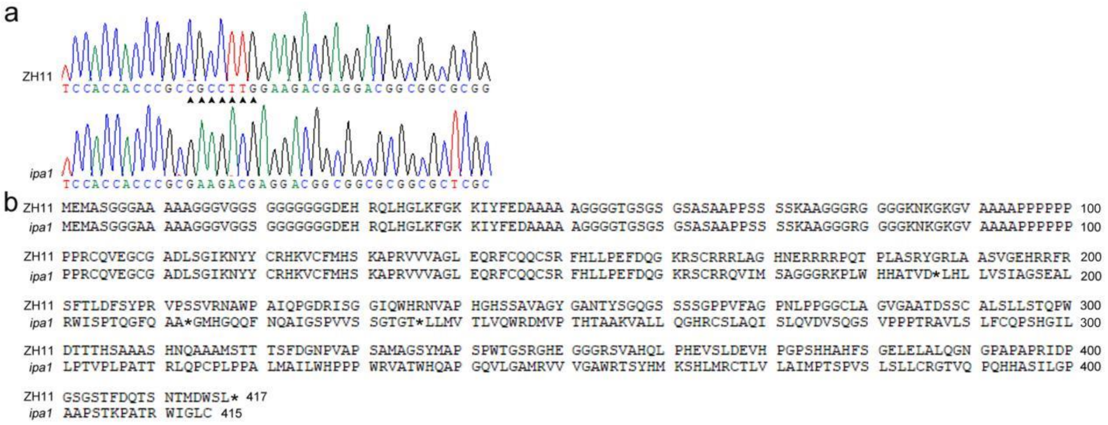

Figure 4. Sequence profiles of the *OsIPA1* gene in the rice mutant *ipa1*

(a, b) The *OsIPA1* gene was knocked out in the rice line Zhonghua 11. Partial gene sequencing profiles (a) and amino acid sequences (b) of the *OsIPA1* gene. Arrowheads indicate deletions of nucleotides in the *OsIPA1* gene. ZH11 = Zhonghua 11. Asterisks

indicate translation termination.

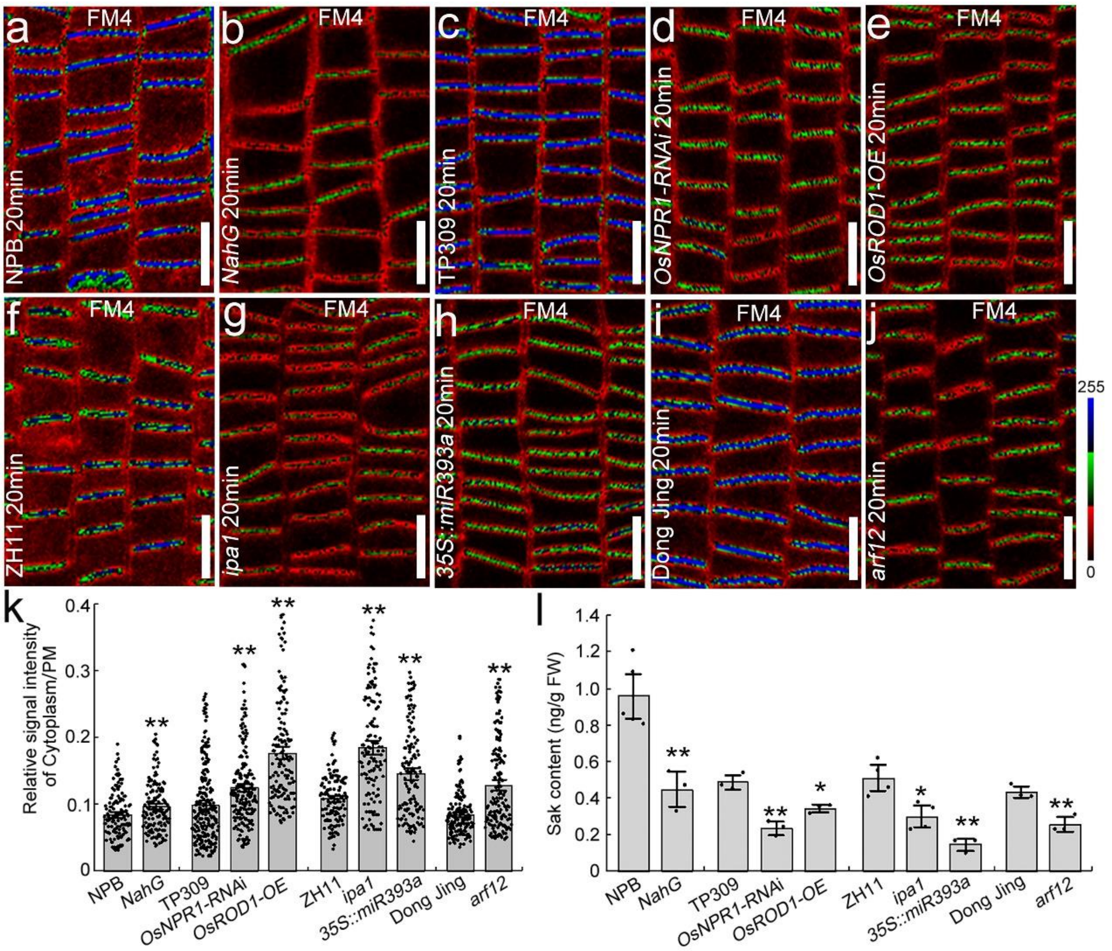

Figure 5. Endocytosis of rice root cells is promoted in the susceptible rice lines

(a-j) Root epidermal cells of 6-day old wild-type rice lines NPB (a), TP309 (c), ZH11 (f), Dong Jing (i) and susceptible rice lines *NahG* (b), *OsNPR1-RNAi* (d), *OsROD1-OE* (e), *ipa1*(g), *35S::miR393a* (h), and *arf12* (j) were labelled with 4 μM FM4-64 for 90 minutes. (k) Quantification of relative fluorescence intensities of plasma membrane versus cytoplasm in rice root epidermal cells shown in the images a-j (n_{NPB} = 131, n_{NahG} = 146, n_{TP309} = 203, $n_{OsNPR1\text{-}RNAi}$ = 186, $n_{OsROD1\text{-}OE}$ = 145, n_{ZH11} = 126, n_{ipa1} = 127, $n_{35S::miR393a}$ = 140, $n_{\text{Dong Jing}}$ = 152 n_{arf12} = 161). (l) Sakuranetin levels in the roots of wild-type rice lines NPB (a), TP309 (c), ZH11 (f), Dong Jing (i), and

susceptible rice lines *NahG* (b), *OsNPR1-RNAi* (d), *OsROD1-OE* (e), *ipa1*(g), *35S::miR393a* (h), and *arf12* (j) (n_{NPB} = 5, n_{NahG} = 3, n_{TP309} = 3, $n_{OsNPR1\text{-}RNAi}$ = 3, $n_{OsROD1\text{-}OE}$ = 3, n_{ZH11} = 4, n_{ipa1} = 4, $n_{35S::miR393a}$ = 3, $n_{Dong\ Jing}$ = 3 , n_{arf12} = 3). NPB = Nipponbare, ZH11 = Zhonghua 11. The relative fluorescence intensity is color-coded: red, low; green, medium; and blue, high fluorescence. PM = plasma membrane, FM4 = FM4-64. Sak = sakuranetin. FW = fresh weight. Data are means ± SE; **P* < 0.05, ***P* < 0.01, *P* values were generated using an independent-samples two-sided Student's *t*-test. Scale bar = 10 μm.

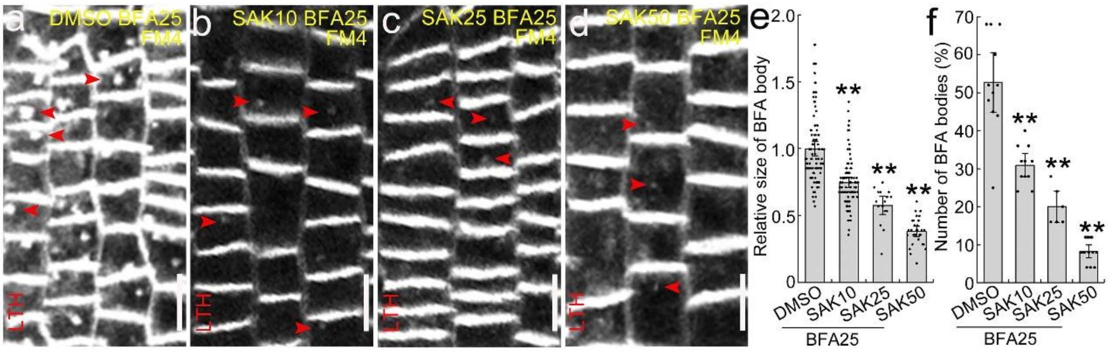

Figure 6. Sakuranetin attenuated endocytosis of rice root cells in a dose-dependent manner

(a-d) The root cells of rice line LTH (a-d) were labelled with 4 μM FM4-64 and co-treated with 25 μM BFA and 10 μM SAK (b), or 25 μM SAK (c), or 50 μM SAK (d), or DMSO as a control (a) for 90 minutes. (e) Quantification of the relative size of the BFA bodies shown in the images a-d (n_{DMSO} = 95;$n_{10\ \mu M\ SAK}$ = 90; $n_{25\ \mu M\ SAK}$ = 16; $n_{50\ \mu M\ SAK}$ = 30). (f) Percentage of BFA bodies in cells shown in the images a-d (n_{DMSO} = 11; $n_{10\ \mu M\ SAK}$ = 11; $n_{25\ \mu M\ SAK}$ = 6; $n_{50\ \mu M\ SAK}$ = 13). LTH = Lijiangxintuanheigu. FM4 = FM4-64, SAK = sakuranetin. Data are means ± SE; ***P* < 0.01, *P* values were

generated using an independent-samples two-sided Student's *t*-test, Scale bar = 10 μm. The arrowheads indicate the BFA bodies.

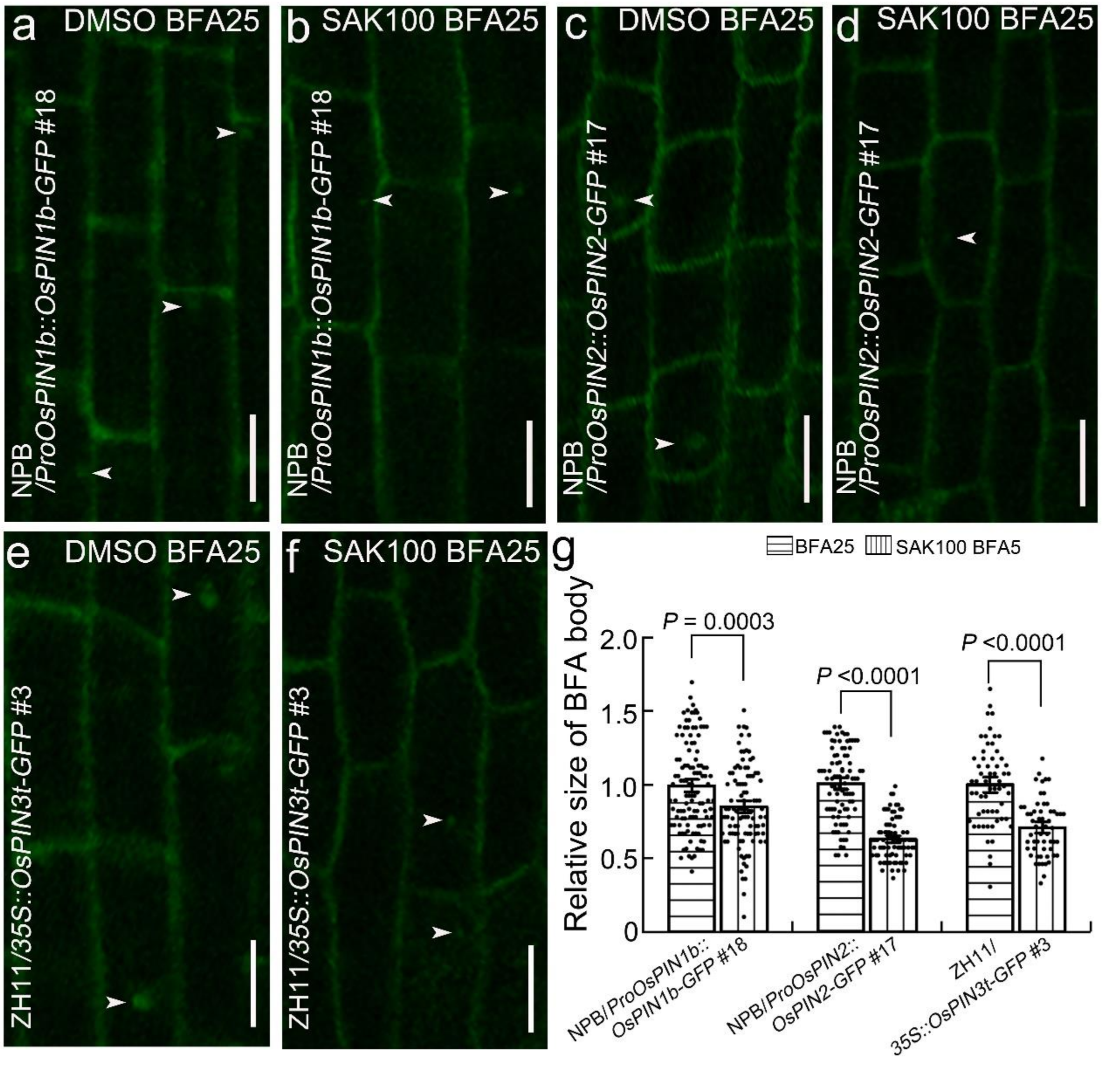

Figure 7. Sakuranetin inhibits endocytosis of the auxin efflux carriers OsPIN1b, OsPIN2 and OsPIN3t

(a-f) The root epidermal cells of the transgenic rice lines NPB/*ProOsPIN1b::OsPIN1b-GFP* (#18) (a, b), NPB/*ProOsPIN2::OsPIN2-GFP* (#17) (c, d) and ZH11/*35S::OsPIN3t-GFP* (#3) (e, f) co-treated for 90 minutes with 100 μM SAK and 25 μM BFA (b, d, f), or with 25 μM BFA and an equivalent volume

of DMSO as a control (a, c, e). (g) Quantification of the relative size of the BFA bodies shown in the images a-f (DMSO: $n_{NPB/ProOsPIN1b::OsPIN1b\text{-}GFP}$ = 114, $n_{NPB/ProOsPIN2::OsPIN2\text{-}GFP}$ = 89, $n_{ZH11/35S::OsPIN3t\text{-}GFP}$ = 60; SAK: $n_{NPB/ProOsPIN1b::OsPIN1b\text{-}GFP}$ = 99, $n_{NPB/ProOsPIN2::\ OsPIN2\text{-}GFP}$ = 81, $n_{ZH11/35S::OsPIN3t\text{-}GFP}$ = 56) . NPB = Nipponbare, ZH11 = Zhonghua 11, SAK = sakuranetin. Data are means ± SE; *P* values were analyzed by using an independent-samples two-sided student's *t*-test in image g. Scale bar = 10 μm. Arrowheads indicate the BFA bodies.

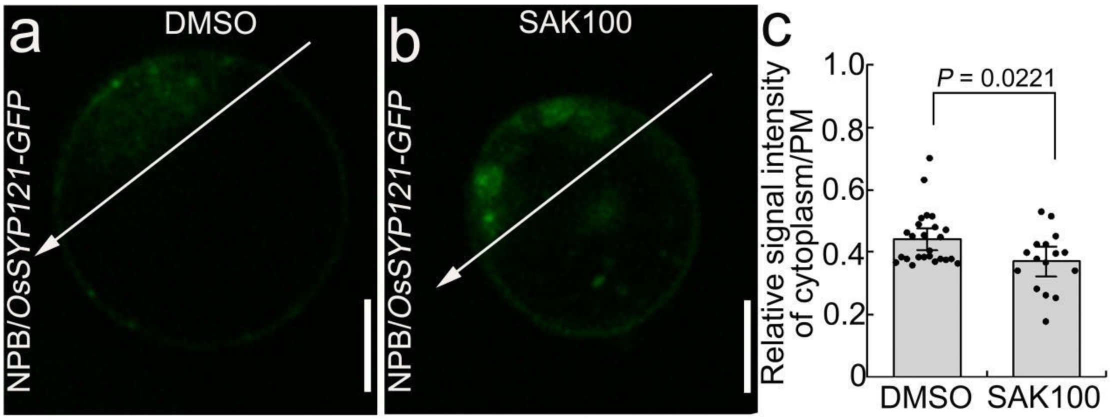

Figure 8. Sakuranetin decreases endocytosis of rice protoplast cells expressing *OsSYP121-GFP*

(a, b) Rice Nipponbare protoplast cells expressing *OsSYP121-GFP* were treated with 100 μM SAK (b) or an equivalent volume of DMSO as a control (a) for 45 minutes. (c) Quantification of fluorescence intensity in the plasma membrane versus the cytoplasm shown in the images a-b (n_{DMSO} = 25, n_{SAK100} = 16). SAK = sakuranetin. Data are means ± SE. *P* values were analyzed using an independent-samples two-sided student's *t*-test (in image c). Scale bar = 5 μm. Arrows indicate the direction of measurement of fluorescence intensity in the plasma membrane and the cytoplasm.

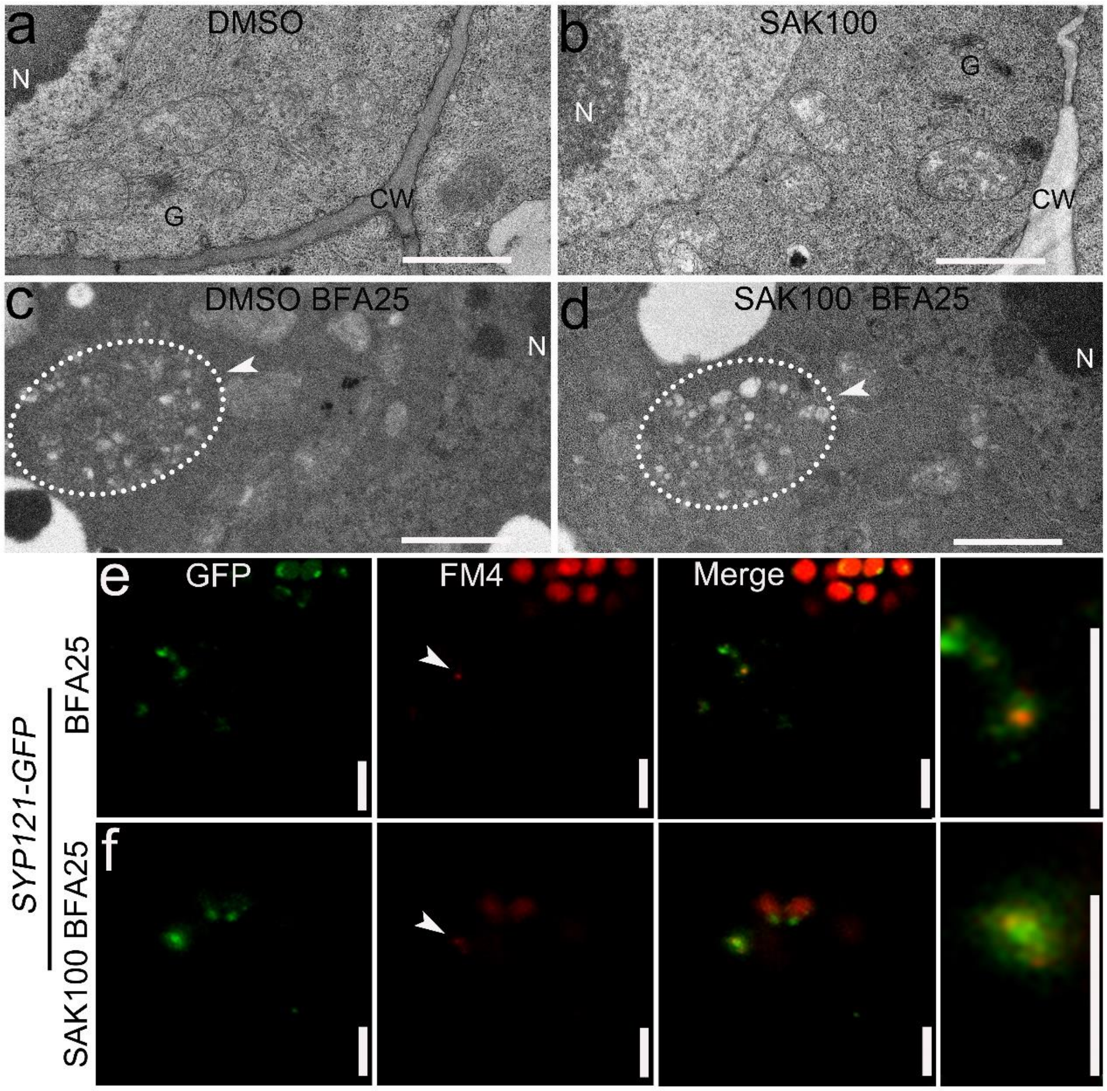

Figure 9. Sakuranetin does not eliminate BFA-induced endosome aggregations

(a-d) Transmission electron micrographs of the cellular ultrastructure of root meristem cells of the rice wild-type Nipponbare treated for 90 minutes with 100 μM SAK (n = 75 cells) (b) or an equivalent volume of DMSO (n = 84 cells) (a), with 25 μM BFA and 100 μM sakuranetin (n = 19 cells) (d) or with 25 μM BFA and an equivalent volume of DMSO (n = 27 cells) (c). (e-f) The rice Nipponbare protoplast cells expessing *SYP121-GFP* (e, f) were labelled with 4 μM FM4-64 and treated with either 25 μM BFA and 100 μM sakuranetin (n = 45 cells) (f) or an equivalent volume

of DMSO as a control (n = 23 cells) (e) for 30 min. SAK = sakuranetin, N = Nucleus, CW = Cell wall, G = Golgi body, Scale bar = 1 μm (in images a-d) or 5 μm (in images e-f). Arrowheads indicate the BFA bodies.

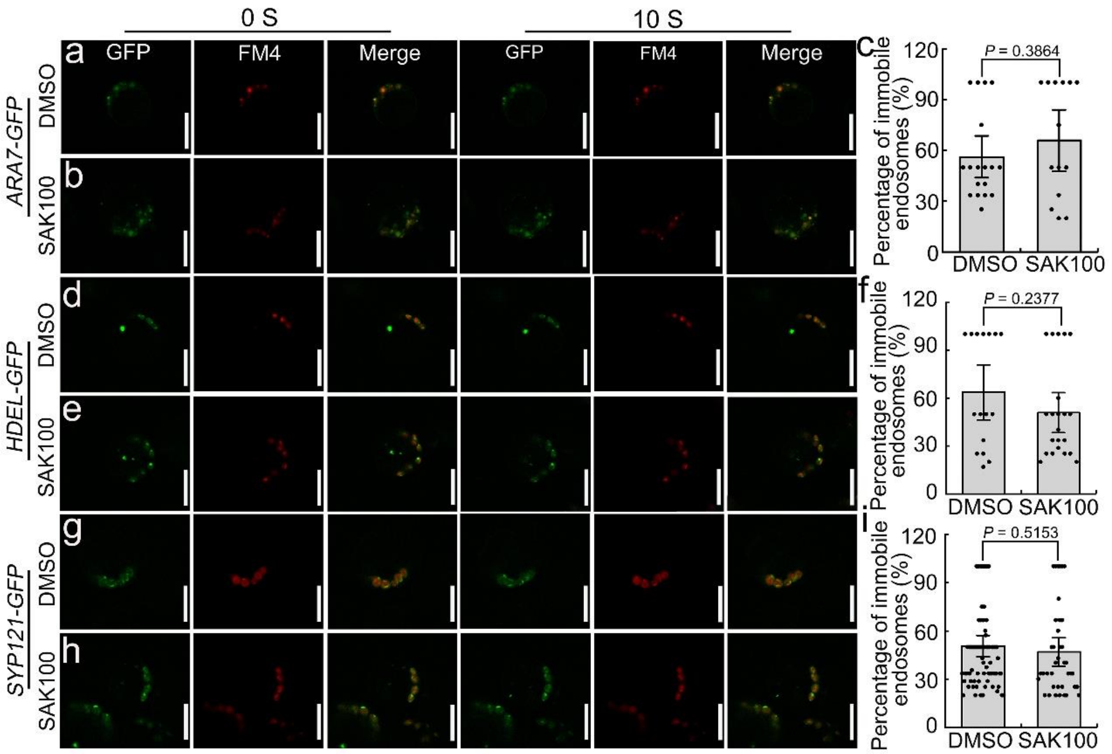

Figure 10. Sakuranetin treatment did not affect endosomal motility

(a, b, d, e, g, h) Rice Nipponbare protoplast cells expressing *ARA7-GFP* (a, b), *HDEL-GFP* (d, e) and *SYP121-GFP* (g, h), all of which label endosomes, were treated with 100 μM SAK (b, e, h) or an equivalent volume of DMSO as a control (a, d, g) for 45 minutes. Images represent sequential imaging at 0 or 10 seconds after the indicated treatment. (c, f, i) Quantifications of the mean percentages of immobile endosomes shown in the images a, b, d, e, g, h (DMSO: $n_{ARA7\text{-}GFP}$ = 18, $n_{HDEL\text{-}GFP}$ = 16, $n_{SYP121\text{-}GFP}$ = 80; SAK100: $n_{ARA7\text{-}GFP}$ = 14, $n_{HDEL\text{-}GFP}$ = 22, $n_{SYP121\text{-}GFP}$ = 39). Merged green and red images were exposed to yellow immobile endosomes at 0 or 10

seconds, respectively, and distinct red and green signals indicate endosomal movement. SAK = sakuranetin, FM4 = FM4-64, Data are means ± SE of the percentage of colocalized immobile vesicles after 10 seconds; *P* values were analyzed using an independent-samples two-sided student's *t*-test in images c, f and i. Scale bar = 5 μm.

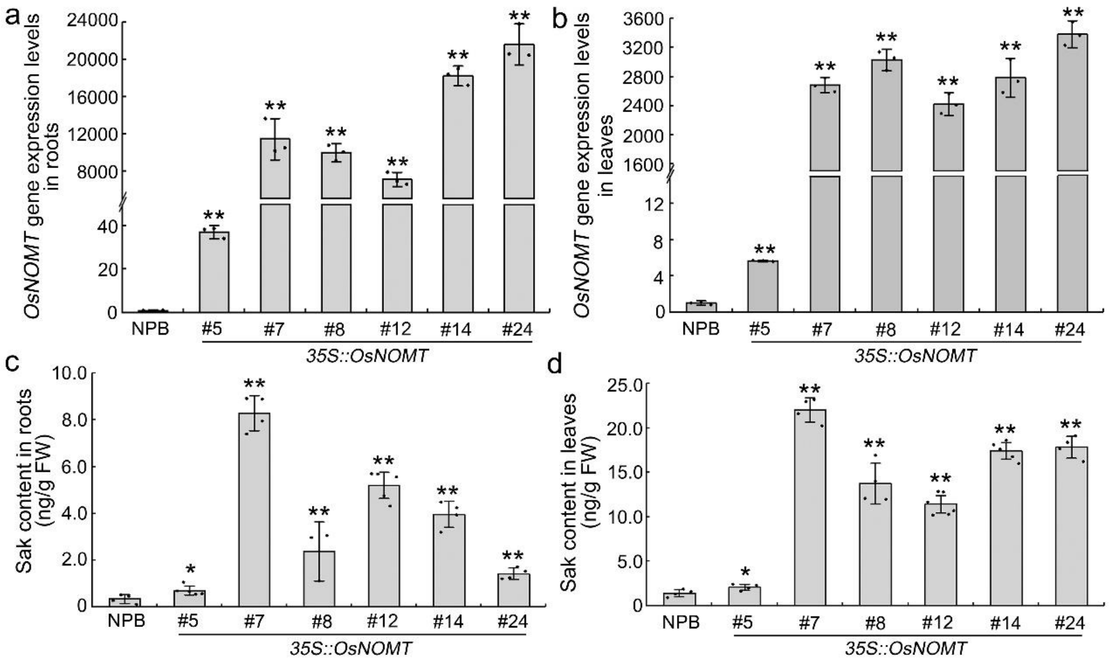

Figure 11. The expression levels of *OsNOMT* in the roots and leaves of transgenic rice lines overexpressing *OsNOMT*

(a-b) Relative expression levels of the *OsNOMT* gene in the roots (a) and leaves (b) of the rice wild-type Nipponbare, and in the transgenic lines #5, #7, #8, #12, #14 and #24 overexpressing *OsNOMT*, assessed using real-time PCR (n = 3 independent experiments). The rice actin gene was used as an internal control. (c-d) Sakuranetin levels in the roots of rice lines overexpressing *OsNOMT* (c) ($n_{NPB} = 4$; $n_{\#5} = 5$; $n_{\#7} = 4$; $n_{\#8} = 3$; $n_{\#12} = 5$; $n_{\#14} = 4$; $n_{\#24} = 4$) and leaves (d) ($n_{NPB} = 4$; $n_{\#5} = 4$; $n_{\#7} = 4$; $n_{\#8} = 4$;

$n_{\#12} = 6$; $n_{\#14} = 5$; $n_{\#24} = 4$). NPB = Nipponbare. Sak = sakuranetin. FW = fresh weight. Data are means ± SE; *$P < 0.05$, **$P < 0.01$ (independent-samples two-sided SPSS analysis in images a and b, independent-samples two-sided Student's *t*-test in images c and d).

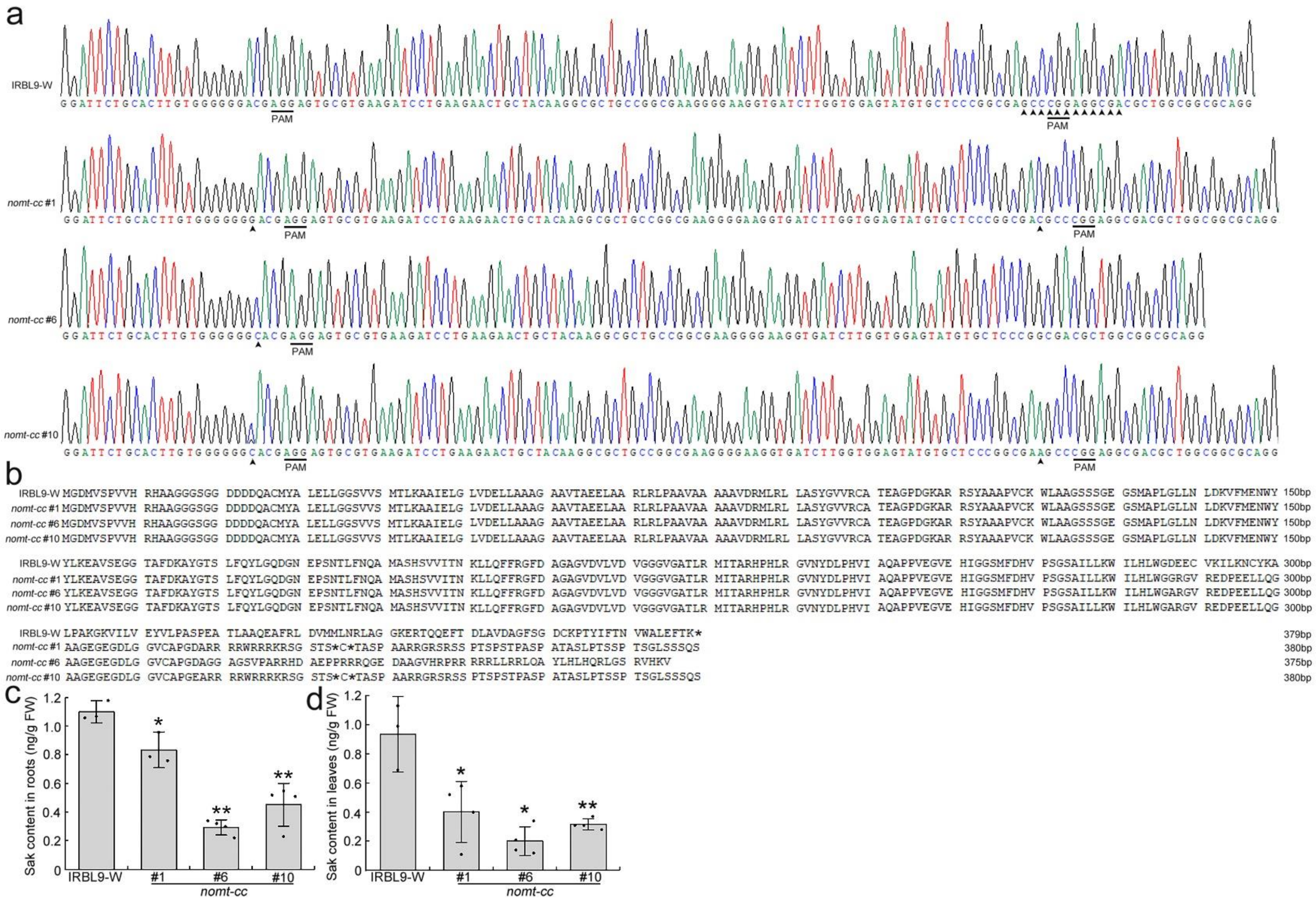

Figure 12. Sequence profiles of the *OsNOMT* gene in the rice lines IRBL9-W and *nomt-cc* mutant

(a-b) The *OsNOMT* gene was knocked out in the rice line IRBL9-W using the CRISPR/Cas9 method. Partial sequences of the *OsNOMT* gene (a) and OsNOMT amino acid (b) in the rice line IRBL9-W and the *nomt*-cc mutant lines #1, #6 and #10. (c-d) Sakuranetin levels in the roots (c) ($n_{\text{IRBL9-W}} = 3$; $n_{\#1} = 3$; $n_{\#6} = 4$; $n_{\#10} = 4$) and leaves of the *nomt*-cc mutant lines (d) ($n_{\text{IRBL9-W}} = 3$; $n_{\#1} = 4$; $n_{\#6} = 4$; $n_{\#10} = 4$). Arrowheads indicate insertions or deletions of nucleotides in the *OsNOMT* gene of *nomt-cc* mutants compared with the rice wild type IRBL9-W. PAM = protospacer adjacent motif. Asterisks indicate translation termination. Sak = sakuranetin. FW = fresh weight. Data are means ± SE; **P* < 0.05, ***P* < 0.01 (independent-samples two-sided Student's *t*-test).

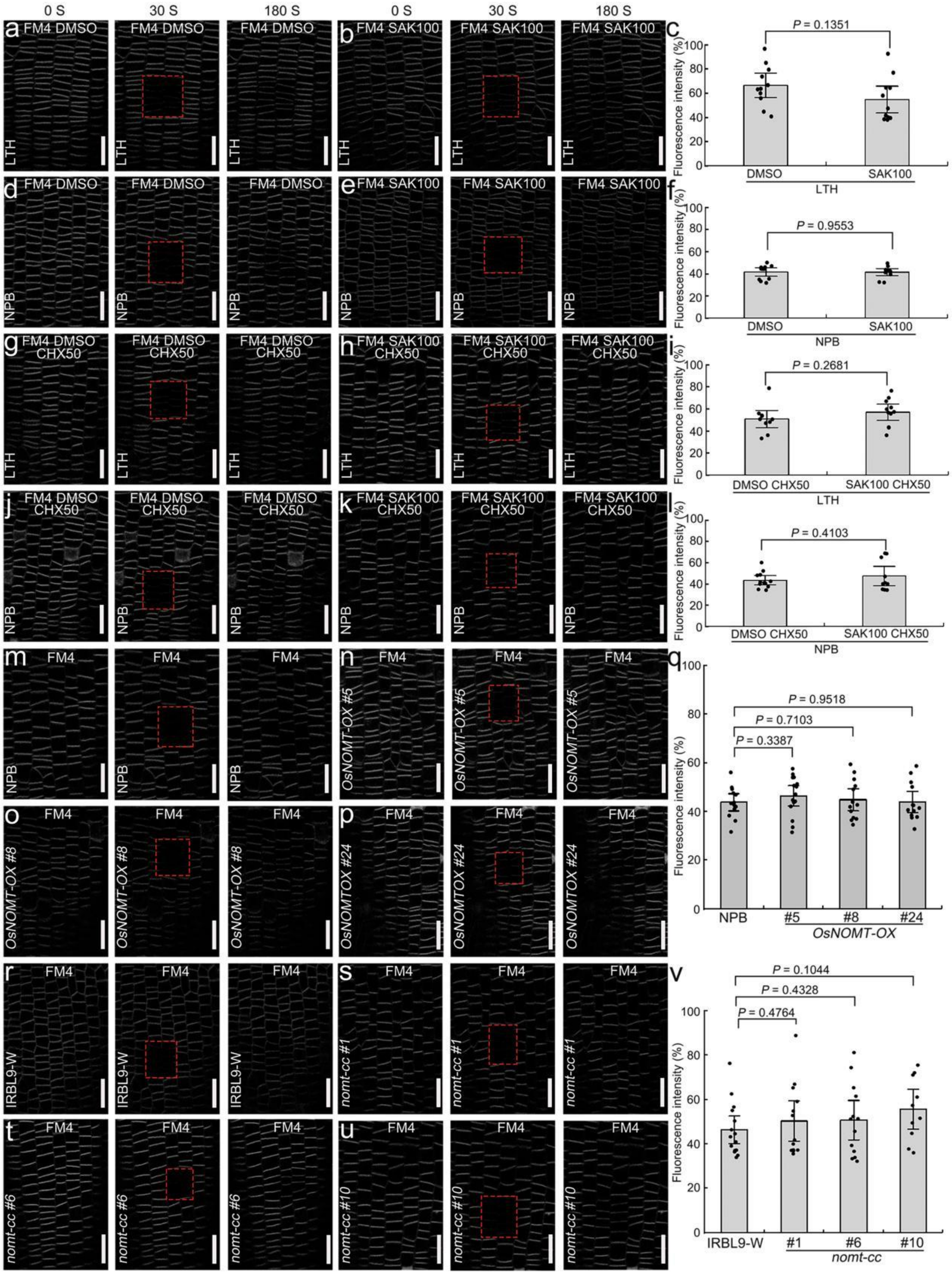

Figure 13. Sakuranetin treatment does not affect the fluidity of the plasma membrane. (a, b, d, e, g, h, j, k) Root epidermal cells of rice lines LTH (a, b, g, h) and Nipponbare (d, e, j, k) were labelled with 4 μM FM4-64 and treated with 100 μM sakuranetin (b, e), and an equivalent volume of DMSO acted as control (a, d), or were treated with

100 μM sakuranetin and 50 μM CHX (h, k), and with 50 μM CHX and an equivalent volume of DMSO as control (g, j) for 90 minutes. (c, f, i, l) The percentage of the recovery of fluorescence intensity after photobleaching is shown in the images a, b, d, e, g, h, j, k (DMSO: $n_{LTH} = 11$, $n_{NPB} = 11$; SAK100: $n_{LTH} = 11$, $n_{NPB} = 11$; CHX50/DMSO: $n_{LTH} = 10$, $n_{NPB} = 12$; CHX50/SAK100: $n_{LTH} = 11$, $n_{NPB} = 10$). (m, n, o, p, r, s, t, u) Root epidermal cells of rice wild-type Nipponbare (m), IRBL9-W (r), lines overexpressing-*OsNOMT* (n-p), and *nomt-cc* mutant lines (s-u) were labelled with 4 μM FM4-64 for 90 minutes. (q, v) The percentage of the recovery of fluorescence intensity after photobleaching is shown in the images m, n, o, p, r, s, t, u ($n_{NPB} = 13$, $n_{\mathit{OsNOMT}\text{-OX } \mathit{\#5}} = 14$, $n_{\mathit{OsNOMT}\text{-OX \#8}} = 13$, $n_{\mathit{OsNOMT}\text{-OX \#24}} = 13$; $n_{\text{IRBL9-W}} = 15$, $n_{\mathit{nomt}\text{-cc } \mathit{\#1}} = 13$, $n_{\mathit{nom}\text{t-cc } \mathit{\#6}} = 13$, $n_{\mathit{nomt}\text{-cc } \mathit{\#10}} = 10$). LTH = Lijiangxintuanheigu, NPB = Nipponbare, *OsNOMT-OX* = rice seedlings expressing *35S::OsNOMT*, CHX = cycloheximide, SAK = sakuranetin. Data are means ± SE; *P* values were generated using independent-samples two-sided Student's *t*-test in images c, f, i, l, q and v. Scale bar = 10 μm. The red boxes indicate the areas of root epidermal cells used in the photobleaching.

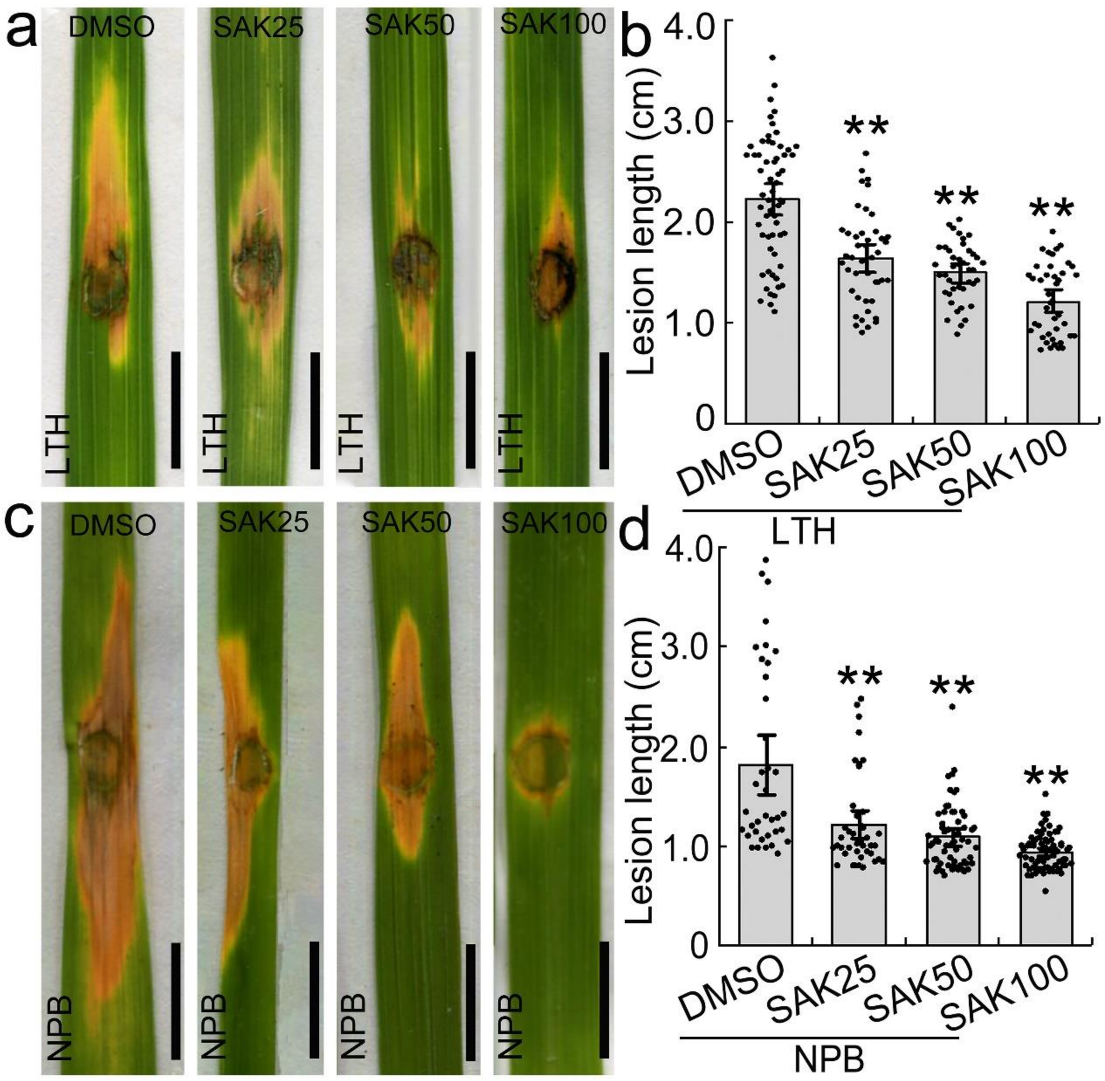

Figure 14. Sakuranetin treatment enhances resistance to rice blast in the lines LTH and NPB

(a, c) Disease lesion phenotype in the leaves of rice lines LTH and NPB inoculated with mycelia of *M. oryzae* Guy11 and treated with 25 μM, 50 μM and 100 μM SAK, or with DMSO as a control. (b, d) Quantification of lesion length shown in the images a and c (DMSO: $n_{LTH} = 60$, $n_{NPB} = 37$; 25 μM SAK: $n_{LTH} = 44$, $n_{NPB} = 41$; 50 μM SAK: $n_{LTH} = 41$, $n_{NPB} = 59$; 100 μM SAK: $n_{LTH} = 42$, $n_{NPB} = 75$). NPB = Nipponbare, LTH = Lijiangxintuanheigu. SAK = sakuranetin. Data are means ± SE; $**P < 0.01$ (independent-samples two-sided Student's *t*-test). Scale bar = 1 cm.

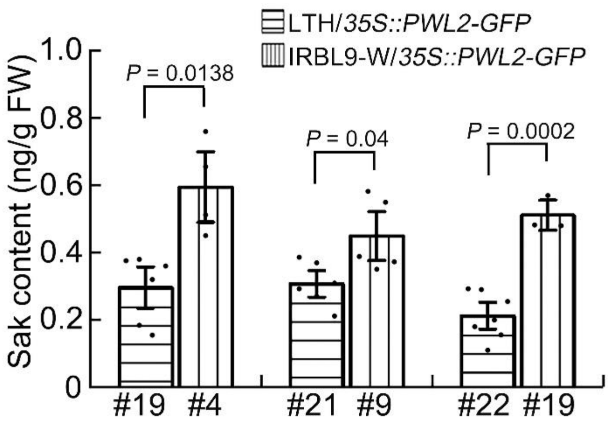

Figure 15. The sakuranetin levels in the rice lines LTH and IRBL9-W expressing the *PWL2-GFP* gene

Sakuranetin levels in the roots of 7-day old rice lines LTH and IRBL9-W expressing *35S::PWL2-GFP* ($n_{LTH/35S::PWL2\text{-}GFP\ \#19}$ = 6; $n_{LTH/35S::PWL2\text{-}GFP\ \#21}$ = 6; $n_{LTH/35S::PWL2\text{-}GFP\ \#22}$ = 7; $n_{IRBL9\text{-}W/35S::PWL2\text{-}GFP\ \#4}$ = 4; $n_{IRBL9\text{-}W/35S::PWL2\text{-}GFP\ \#9}$ = 5; $n_{IRBL9\text{-}W/35S::PWL2\text{-}GFP\ \#19}$ = 3) measured using HPLC-MS/MS. Sak = sakuranetin. FW = fresh weight. Data are means ± SE; *P* values were generated using an independent-samples two-sided Student's *t*-test.

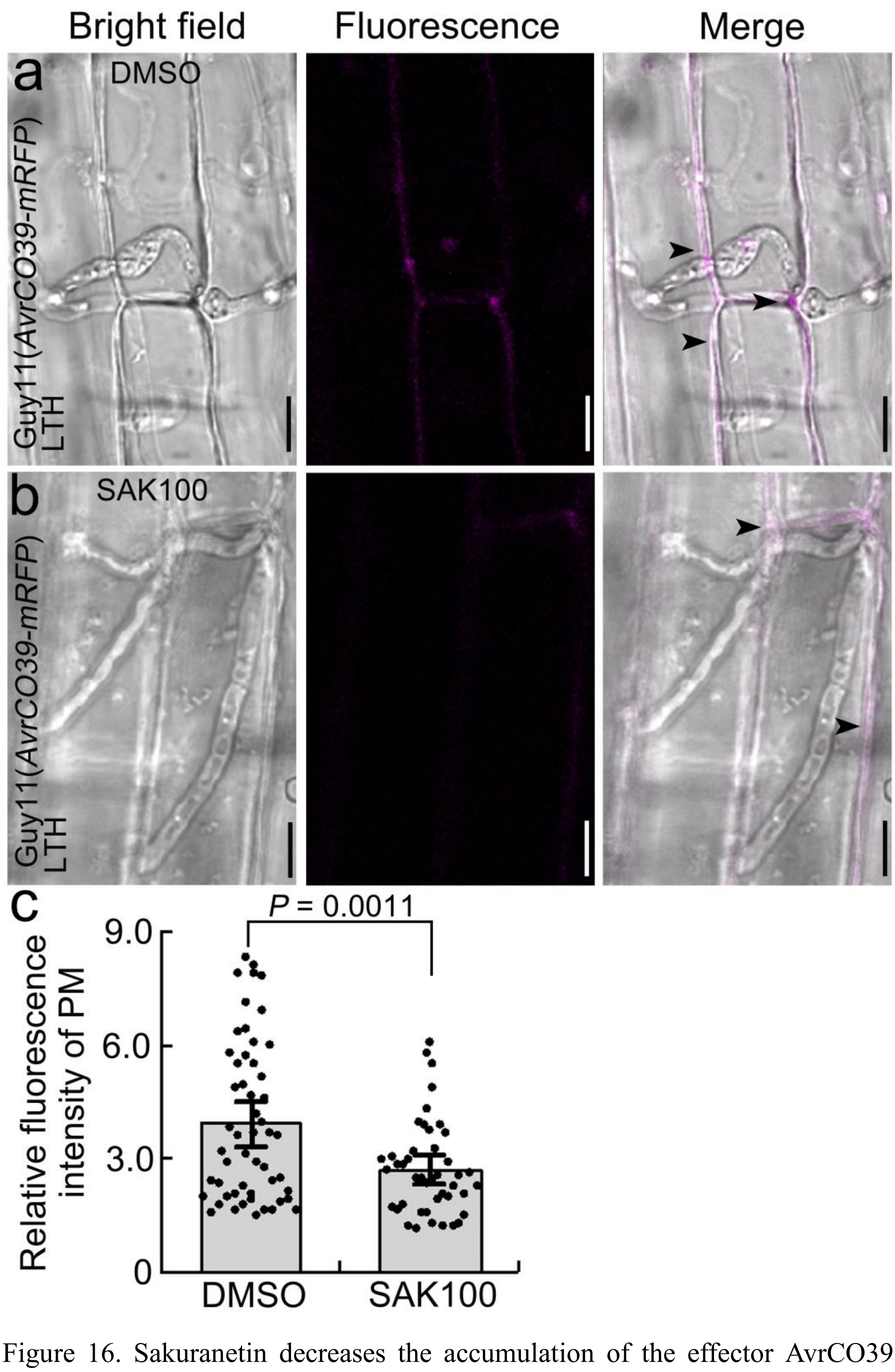

Figure 16. Sakuranetin decreases the accumulation of the effector AvrCO39 of *M. oryzae* in the rice plasma membrane

(a, b) The leaf sheaths of rice seedlings inoculated with a spore suspension of *M. oryzae* strain Guy11 expressing *AvrCo39-mRFP* and treated with 100 μM sakuranetin

(b) or with an equivalent volume of DMSO as a control (a). (c) Quantification of the relative fluorescence intensity of the PM shown in the images a-b (n_{DMSO} = 53, n_{SAK} = 44). LTH = Lijiangxintuanheigu, PM = plasma membrane, SAK = sakuranetin. Data are means ± SE; *P* values were generated using an independent-samples two-sided student's *t*-test in image c. Scale bar = 10 μm. Arrowheads indicate the localization of *AvrCo39-mRFP* protein in the PM of leaf sheath cells.
