# Supplementary material for: Phytoalexin sakuranetin attenuates endocytosis and enhances resistance to rice blast
Source: Nat Commun. 2024 Apr 23;15:3437. doi: 10.1038/s41467-024-47746-y (PMC11039461; doi:10.1038/s41467-024-47746-y)
Supplement: Supplementary file 3 — Reporting Summary [file 41467_2024_47746_MOESM3_ESM.pdf]

nature portfolio

Corresponding author(s): Yunlong Du
Last updated by author(s): Mar 25, 2024

Reporting Summary

Nature Portfolio wishes to improve the reproducibility of the work that we publish. This form provides structure for consistency and transparency in reporting. For further information on Nature Portfolio policies, see our Editorial Policies and the Editorial Policy Checklist.

Statistics

For all statistical analyses, confirm that the following items are present in the figure legend, table legend, main text, or Methods section.

n/a	Confirmed	
☐	☒	The exact sample size (*n*) for each experimental group/condition, given as a discrete number and unit of measurement
☐	☒	A statement on whether measurements were taken from distinct samples or whether the same sample was measured repeatedly
☐	☒	The statistical test(s) used AND whether they are one- or two-sided<br>*Only common tests should be described solely by name; describe more complex techniques in the Methods section.*
☒	☐	A description of all covariates tested
☒	☐	A description of any assumptions or corrections, such as tests of normality and adjustment for multiple comparisons
☐	☒	A full description of the statistical parameters including central tendency (e.g. means) or other basic estimates (e.g. regression coefficient) AND variation (e.g. standard deviation) or associated estimates of uncertainty (e.g. confidence intervals)
☐	☒	For null hypothesis testing, the test statistic (e.g. *F*, *t*, *r*) with confidence intervals, effect sizes, degrees of freedom and *P* value noted<br>*Give P values as exact values whenever suitable.*
☒	☐	For Bayesian analysis, information on the choice of priors and Markov chain Monte Carlo settings
☒	☐	For hierarchical and complex designs, identification of the appropriate level for tests and full reporting of outcomes
☒	☐	Estimates of effect sizes (e.g. Cohen's *d*, Pearson's *r*), indicating how they were calculated

Our web collection on statistics for biologists contains articles on many of the points above.

Software and code

Policy information about availability of computer code

Data collection	Image J 1.41 software, Leica SP8 confocal microscope (TCS SP8), Leica SP5 confocal microscope, LEICA EM UC7 ultramicrotome, FEI TECNAI, FEI TECNAI SPIRIT G2 TEM, HPLC-MS/MS (LCMS-8040)
Data analysis	Student's t test, software SPSS Version 19.0, Origin64

For manuscripts utilizing custom algorithms or software that are central to the research but not yet described in published literature, software must be made available to editors and reviewers. We strongly encourage code deposition in a community repository (e.g. GitHub). See the Nature Portfolio guidelines for submitting code & software for further information.

Data

Policy information about availability of data

All manuscripts must include a data availability statement. This statement should provide the following information, where applicable:

- Accession codes, unique identifiers, or web links for publicly available datasets
- A description of any restrictions on data availability
- For clinical datasets or third party data, please ensure that the statement adheres to our policy

All data supporting the findings of this study are available within the paper, its Supplementary Information file, and the Source Data file. Source data are provided with this paper.

Research involving human participants, their data, or biological material

Policy information about studies with human participants or human data. See also policy information about sex, gender (identity/presentation), and sexual orientation and race, ethnicity and racism.

Reporting on sex and gender	No, we did not use any human participants or human data in this manuscript.
Reporting on race, ethnicity, or other socially relevant groupings	No, this manuscript does not involve in race, ethnicity, or other socially relevant groupings.
Population characteristics	See above
Recruitment	See above
Ethics oversight	See above

Note that full information on the approval of the study protocol must also be provided in the manuscript.

Field-specific reporting

Please select the one below that is the best fit for your research. If you are not sure, read the appropriate sections before making your selection.

[x] Life sciences [ ] Behavioural & social sciences [ ] Ecological, evolutionary & environmental sciences

For a reference copy of the document with all sections, see nature.com/documents/nr-reporting-summary-flat.pdf

Life sciences study design

All studies must disclose on these points even when the disclosure is negative.

Sample size

To analyze the disease index showed in Figure 1l, sample sizes are 8 to 43, the statistical method is Student's t test.
To analyze the fluorescence intensities showed in Figure 1x, sample sizes are 53 to 194, the statistical method is Student's t test.
To analyze the fluorescence intensities showed in Figure 2e, sample sizes are 18 to 100, the statistical method is Student's t test.
To analyze the relative size of the BFA bodies showed in Figure 2j, 2s and 2y, sample sizes are 8 to 244, the statistical method is Student's t test.
To analyze the number of BFA bodies showed in Figure 2k, 2t and 2z, sample sizes are 5 to 26, the statistical method is Student's t test.
To analyze the disease index showed in Figure 2ab and 2ae, sample sizes are 11 to 72, the statistical method is Student's t test.
To analyze the relative size of the BFA bodies showed in Figure 3g, 3j, 3o and 3s, sample sizes are 15 to 188, the statistical method is Student's t test.
To analyze the disease index showed in Figure 4b, and 4e, sample sizes are 31 to 79, the statistical method is Student's t test.
To analyze the lesion length showed in Figure 4h, sample sizes are 64 to 102, the statistical method is Student's t test.
To analyze the sakuranetin levels showed in Supplementary Figure 1, sample sizes are 3 to 6, the statistical method is Student's t test.
To analyze the relative size of the BFA bodies showed in Supplementary Figure 2l, sample sizes are 59 to 283, the statistical method is Student's t test.
To analyze the number of BFA bodies showed in Supplementary Figure 2m, sample sizes are 5 to 18, the statistical method is Student's t test.
To analyze the sakuranetin levels showed in Supplementary Figure 2n, sample sizes are 3 to 8, the statistical method is SPSS analysis.
To analyze the fluorescence intensity showed in Supplementary Figure 3c, sample sizes are 90 to 150, the statistical method is Student's t test.
To analyze the relative size of the BFA bodies showed in Supplementary Figure 3f, sample sizes are 151 to 182, the statistical method is Student's t test.
To analyze the number of BFA bodies showed in Supplementary Figure 3g, sample sizes are 13, the statistical method is Student's t test.
To analyze the sakuranetin levels showed in Supplementary Figure 3h, sample sizes are 4, the statistical method is Student's t test.
To analyze the fluorescence intensity showed in Supplementary Figure 5k, sample sizes are 126 to 203, the statistical method is Student's t test.
To analyze the sakuranetin levels showed in Supplementary Figure 5l, sample sizes are 3 to 5, the statistical method is Student's t test.
To analyze the relative size of the BFA bodies showed in Supplementary Figure 6e, sample sizes are 16 to 95, the statistical method is Student's t test.
To analyze the number of BFA bodies showed in Supplementary Figure 6f, sample sizes are 6 to 13, the statistical method is Student's t test.
To analyze the relative size of the BFA bodies showed in Supplementary Figure 7g, sample sizes are 56 to 114, the statistical method is Student's t test.
To analyze the fluorescence intensity showed in Supplementary Figure 8c, sample sizes are 16 to 25, the statistical method is Student's t test.
To analyze the BFA-induced endosome aggregations showed in Supplementary Figure 9a, 9b, 9c, 9d, 9e and 9f, number of the cells are 19 to 84.
To analyze the immobile endosomes showed in Supplementary Figure 10c, 10f and 10i, sample sizes are 14 to 80, the statistical method is Student's t test.
To analyze the sakuranetin levels showed in Supplementary Figure 11c and 11d, sample sizes are 3 to 6, the statistical method is SPSS analysis.
To analyze the sakuranetin levels showed in Supplementary Figure 12c and 12d, sample sizes are 3 to 4, the statistical method is Student's t test.
To analyze the fluorescence intensity showed in Supplementary Figure 13c, 13f, 13i, 13l, 13q and 13v, sample sizes are 10 to 15, the statistical method is Student's t test.

	To analyze the lesion length showed in Supplementary Figure 14b and 14d, sample sizes are 37 to 75, the statistical method is Student's t test.<br>To analyze the sakuranetin levels showed in Supplementary Figure 15, sample sizes are 3 to 7, the statistical method is SPSS analysis.<br>To analyze the fluorescence intensity showed in Supplementary Figure 16c, sample sizes are 44 to 53, the statistical method is Student's t test.<br>To analyze the expression levels of the MoPot2 gene (Figure 2ac, 2af, 4c, 4f and 4i) and the OsNOMT gene (Figure S11a and S11b), biologically independent experiments are 3, the statistical method is SPSS analysis.
Data exclusions	In this text, some data from disease index (Figure 1i, 2ab, 2ae, 4b, 4e), lesion length (Figure 4h, S14b, S14d), fluorescence intensities (Figure 1x, 2e, S3c, S5k, S8c, S13c, S13f, S13i, S13l, S13q, S13v, S16c), relative size of the BFA bodies (Figure 2j, 2s, 2y, 3g, 3j, 3o, 3s, 4c, 4f, 4i, S2l, S3f, S6e, S7g), number of the BFA bodies (Figure 2k, 2t, 2z, S3g, S2m, S6f), sakuranetin content (Figure S1, S2n, S3h, S5l, S11c-d, S12c-d, S15) and percentages of immobile endosomes (Figure S10c, S10f, S10i) were excluded from the analyses, as these excluded data showed significant deviations, the values are either too high or too low, and we did not pre-established exclusion criteria. For the data of gene expression level (Figure 2ac, 2af, 4c, 4f, 4i, S11a-b), no data were excluded from the analyses.
Replication	To verify the reproducibility of the experimental findings, each experiment was developed at least three replicates, and all attempts at replication were successful.
Randomization	Samples were randomly allocated into different experimental groups, and two or three participants were randomly allocated into every experiment to develop different works.
Blinding	Two or three investigators were blinded to group allocation during data collection and analysis.

Reporting for specific materials, systems and methods

We require information from authors about some types of materials, experimental systems and methods used in many studies. Here, indicate whether each material, system or method listed is relevant to your study. If you are not sure if a list item applies to your research, read the appropriate section before selecting a response.

Materials & experimental systems

n/a	Involved in the study
☒	☐ Antibodies
☒	☐ Eukaryotic cell lines
☒	☐ Palaeontology and archaeology
☒	☐ Animals and other organisms
☒	☐ Clinical data
☒	☐ Dual use research of concern
☐	☒ Plants

Methods

n/a	Involved in the study
☒	☐ ChIP-seq
☒	☐ Flow cytometry
☒	☐ MRI-based neuroimaging

Dual use research of concern

Policy information about dual use research of concern

Hazards

Could the accidental, deliberate or reckless misuse of agents or technologies generated in the work, or the application of information presented in the manuscript, pose a threat to:

No	Yes	
☒	☐	Public health
☒	☐	National security
☒	☐	Crops and/or livestock
☒	☐	Ecosystems
☒	☐	Any other significant area

Experiments of concern

Does the work involve any of these experiments of concern:

No	Yes	
☒	☐	Demonstrate how to render a vaccine ineffective
☒	☐	Confer resistance to therapeutically useful antibiotics or antiviral agents
☒	☐	Enhance the virulence of a pathogen or render a nonpathogen virulent
☒	☐	Increase transmissibility of a pathogen
☒	☐	Alter the host range of a pathogen
☒	☐	Enable evasion of diagnostic/detection modalities
☒	☐	Enable the weaponization of a biological agent or toxin
☒	☐	Any other potentially harmful combination of experiments and agents

Plants

Seed stocks

In this text, the transgenic rice line expressing the Pigm gene, OsNPR1-RNAi mutant line and ROD1-overexpression rice line were obtained from Professor Zhuhua He (Chinese Academy of Sciences. The rice line NahG was obtained from Professor Yinong Yang (The Pennsylvania State University). The transgenic plants overexpressing miR393a was obtained from Professor Muyuan Zhu (Zhejiang University). The arf12 mutant line was obtained from Professor Yanhua Qi (Zhejiang University). The rice NILs lines was obtained from International Rice Research Institute (IRRI). The rice line overexpressing OsPIN3t-GFP (in a Zhonghua 11 background) was obtained from Professor Zhengge Zhu (Hebei Normal University). The rice lines 35S::OsNOMT (in a Nipponbare background), ProOsPIN1b::OsPIN1b-GFP (in a Nipponbare background), ProOsPIN2::OsPIN2-GFP (in a Nipponbare background), 35S::PWL2-GFP (expressing in a LTH and IRBL9-W background, respectively), chc-cc (in a Nipponbare background), nomt-cc (in a IRBL9-W background) and ipa1 (in a Zhonghua 11 background) were constructed at Biogle Company (Hangzhou Biogle Co., Ltd, Hangzhou, China).

Novel plant genotypes

All rice seedlings were grown in laboratory greenhouse and did not spread outside field, and one person responsible for the management of utilization and collection of rice seeds. We isolated the genomic DNA from the rice mutant lines nomt-cc and ipa1 and amplified the target sequences by PCR method. We found that there were deletions of nucleotides in the target site of OsIPA1 gene of ipa1 mutant (Figure S4) and insertions or deletions of nucleotides in the target site of OsNOMT gene of nomt-cc mutants (Figure S12a-b) by sequencing the PCR product.
We observed rice endocytosis ([illegible]), checked rice resistance to rice blast (Figure 2a-f) and measured sakuranetin level (Figure S12c-d) and fluidity of the plasma membrane (Figure S13s-v) using the nomt-cc mutant.
We observed rice endocytosis (Figure S5g, 5k) and measured sakuranetin level (Figure S5l) using the ipa1 mutant.
We did not find off-target gene editing in the nomt-cc and ipa1 mutants by sequencing PCR product.

Authentication

In this text, the rice lines 35S::OsNOMT (in a Nipponbare background) and 35S::PWL2-GFP (expressing in a LTH and IRBL9-W background, respectively) were obtained using the Agrobacterium-mediated method by the Biogle Company (Hangzhou Biogle Co., Ltd, Hangzhou, China).
The T2 rice Nipponbare lines (#5, #7, #8, #12, #14 and #24) expressing 35S::OsNOMT were used to observed rice endocytosis (Figure 2m-r) and measure sakuranetin levels (Figure S11c-d).
The T3 rice LTH lines (#19, #21 and #22) and IRBL9-W lines (#4, #9 and #19) expressing 35S::PWL2-GFP were used to observed rice endocytosis (Figure 3a-j) and measure sakuranetin levels (Figure S15).
The rice mutant lines nomt-cc (with an IRBL9-W background) and ipa1 (with a Zhonghua 11 background) were produced using the CRISPR/Cas9 method at Biogle Company (Hangzhou Biogle Co., Ltd, Hangzhou, China). The specific guide-RNA (gRNA) sequences for OsNOMT and OsIPA1 were designed as follows: 5’-TCTGCACTTGTGGGGGGACG-3’ and 5’-GTATGTGCTCCCGGCGAGCC-3’ for the OsNOMT gene and 5’-GCCGTCCTCGTCTTCCAAGG -3’ for the OsIPA1 gene. The target gene sequences including the protospacer adjacent motif (PAM) of OsNOMT were 5’-TCTGCACTTGTGGGGGGACGAGG-3’ and 5’-GTATGTGCTCCCGGCGAGCCCGG-3’ for the OsNOMT gene and 5’-GCCGTCCTCGTCTTCCAAGGCGG-3’ for the OsIPA1 gene, and the trinucleotides AGG and CGG for the OsNOMT gene and CGG for the OsIPA1 gene acted as the PAMs of nomt-cc and ipa1, respectively.
